# Thermal stability of stealth and de Sitter spacetimes in scalar-tensor gravity

**DOI:** 10.1140/epjc/s10052-023-11697-3

**Published:** 2023-07-15

**Authors:** Serena Giardino, Andrea Giusti, Valerio Faraoni

**Affiliations:** 1 Max Planck Institute for Gravitational Physics (Albert Einstein Institute), Callinstraße 38, 30167 Hannover, Germany; 2 Institute for Theoretical Physics, Heidelberg University, Philosophenweg 16, 69120 Heidelberg, Germany; 3 Institute for Theoretical Physics, ETH Zurich, Wolfgang-Pauli-Strasse 27, 8093 Zurich, Switzerland; 4 Department of Physics and Astronomy, Bishop’s University, 2600 College Street, Sherbrooke, QC J1M 1Z7 Canada

## Abstract

Stealth solutions of scalar-tensor gravity and less-known de Sitter spaces that generalize them are analyzed regarding their possible role as thermal equilibria at non-zero temperature in the new first-order thermodynamics of scalar-tensor gravity. No stable equilibria are found, further validating the special role of general relativity as an equilibrium state in the landscape of gravity theories, seen through the lens of first-order thermodynamics.

## Introduction

A surprising and intriguing relationship appears to exist between thermodynamics and gravitation. Two seminal works showed that both the Einstein equations of general relativity (GR) and the field equations of metric *f*(*R*) gravity can be recovered from purely thermodynamical considerations, starting with a few assumptions [1, 2]. However, dealing with a modified theory of gravity requires a generalization to a non-equilibrium thermodynamical setting. These works put forward the idea that, in the landscape of gravity theories, GR could be an equilibrium state and modified gravity a non-equilibrium one. This idea was made more concrete by the recent proposal [3, 4] of a first-order thermodynamics of scalar-tensor theories, with minimal assumptions and in a context completely different from that of spacetime thermodynamics [1, 2]. Scalar-tensor theories represent prototypical candidates of modified gravity and were first introduced by Brans and Dicke in [5] and then extended in [6–8]. The first-order thermodynamical proposal relies on interpreting the scalar contributions as an imperfect fluid [9–11] and applying a non-equilibrium thermodynamical description [12] to it. This idea unexpectedly allows one to introduce a concept of “temperature of gravity” (which is clearly no physical temperature, but simply a temperature relative to the GR equilibrium state) and an understanding of the dissipative process leading gravity towards (or away from) the GR state of equilibrium. This proposition has been applied and tested on both different classes of theories (such as Horndeski gravity) and specific solutions of scalar-tensor theories, such as those in Friedmann–Lemaître–Robertson–Walker (FLRW) spacetime [13, 14].

A characteristic feature of scalar-tensor gravity is the existence of stealth solutions, namely solutions with the same geometry of GR solutions but with a nontrivial scalar field profile that does not contribute to the effective stress-energy tensor. Current motivation to study stealth solutions comes from the possibility of detecting black hole hair in stealth black holes through gravitational wave observations [15]. Indeed, “first-generation” scalar-tensor and Horndeski theories allow for stealth solutions that violate some assumptions of the no-hair theorems and for which the scalar field does not gravitate. This would in principle make it possible to observationally distinguish GR from scalar-tensor theories. Such solutions include stealth Schwarzschild (-de Sitter) black holes with a scalar field linearly dependent on time in the context of Horndeski and beyond-Horndeski gravity [16–21].

Here we are interested in stealth solutions in the framework of scalar-tensor thermodynamics, where they would correspond to different “states of gravity” away from the GR equilibrium, as explained in the following. Studying these solutions would therefore help to clarify the existence of equilibrium states different from GR and establish which gravity theories or specific solutions could approach them, extending the study of scalar-tensor thermodynamics to uncharted territory. Assessing the stability of such states is crucial: it is reasonable to expect that, due to the special status of the GR equilibrium state in the landscape of gravity theories, these other equilibria would be unstable, thus less relevant than GR.

The stability of certain stealth geometries has been previously studied with the Bardeen–Ellis–Bruni–Hwang [22–26] approach for cosmological perturbations in modified gravity [27–32]. Here, we propose a complementary criterion based solely on our thermodynamical formalism. Insights coming from thermodynamics provide essential guidance to both approaches, as for the stealth spacetime studied in [33] with the gauge-invariant formalism. In that case, stability was assessed with the gauge-invariant criterion, while in the present work we mostly use the thermal criterion.

Stealth solutions include those of Refs. [15, 34–46]. Often these are degenerate cases of de Sitter spaces with non-constant scalar fields, which are not as well-known as stealth solutions of the field equations. de Sitter spaces with constant scalar fields are fixed points of the dynamical system of scalar-tensor cosmology [47] and are also common in GR cosmology sourced by scalar fields. On the contrary, de Sitter spaces with a non-constant scalar field are a signature of modified gravity. Both stealth solutions and de Sitter universes with non-constant scalar fields seem peculiar and deserve investigation in the thermodynamics of scalar-tensor gravity.

We follow the notation of Ref. [48]. The (Jordan frame) scalar-tensor action reads

1 $$\begin{aligned} S_\textrm{ST}= & {} \frac{1}{16\pi } \int d^4x \sqrt{-g} \left[ \phi R -\frac{\omega (\phi )}{\phi } \, \nabla ^c\phi \nabla _c\phi -V(\phi ) \right] \nonumber \\ {}{} & {} +S^\mathrm {(m)} \,, \end{aligned}$$

where *R* is the Ricci scalar, the Brans–Dicke scalar $\phi >0$ is approximately the inverse of the effective gravitational coupling $G_\textrm{eff}$, $\omega (\phi )$ is the “Brans–Dicke coupling”, $V(\phi )$ is the scalar field potential, and $S^\mathrm {(m)}=\int d^4x \sqrt{-g} \, \mathcal {L}^\mathrm {(m)}$ is the matter action. The field equations are [5–8]

2 $$\begin{aligned} R_{ab} - \frac{1}{2}\, g_{ab} R&= \frac{8\pi }{\phi } \, T_{ab}^\mathrm {(m)}+ \frac{\omega }{\phi ^2} \left( \nabla _a \phi \nabla _b \phi -\frac{1}{2} \, g_{ab} \nabla _c \phi \nabla ^c \phi \right) \nonumber \\&\quad +\frac{1}{\phi } \left( \nabla _a \nabla _b \phi - g_{ab} \Box \phi \right) -\frac{V}{2\phi }\, g_{ab} \,, \end{aligned}$$

3 $$\begin{aligned} (2\omega +3) \, \Box \phi&= \left( 8\pi T^\mathrm {(m)}+ \phi \, V_{,\phi } -2V -\omega _{,\phi } \nabla ^c \phi \nabla _c \phi \right) \,, \end{aligned}$$

where $R_{ab}$ is the Ricci tensor, $T^\mathrm {(m)} \equiv g^{ab}T_{ab}^\mathrm {(m)}$ is the trace of the matter stress-energy tensor $T_{ab}^\mathrm {(m)}$, $\omega _{,\phi } \equiv d\omega /d\phi$ and $V_{,\phi } \equiv dV/d\phi$.

## Thermal stability criterion

Assuming $\nabla ^a \phi$ to be timelike and future-oriented, it is used to define the four-velocity of an effective irrotational fluid

4 $$\begin{aligned} u^a = \frac{ \nabla ^a \phi }{ \sqrt{ -\nabla ^c \phi \nabla _c\phi }} \,. \end{aligned}$$

The effective stress-energy tensor of $\phi$ in the effective Einstein equations (2) has the form of a dissipative fluid which, surprisingly, obeys Eckart’s constitutive relations [12], namely the simplest assumptions to satisfy the covariant second law of thermodynamics in a non-equilibrium setting. These relations connect the dissipative quantities to the constitutive variables of the fluid: for example, the heat current density $q_a^{(\phi )}$ is related to the temperature $\mathcal {T}$ with the thermal conductivity coefficient $\mathcal {K}$, through the generalized Fourier law

5 $$\begin{aligned} q_a^{(\phi )} = -\mathcal {K} \left( h_{ab} \nabla ^b \mathcal {T} + \mathcal {T} \dot{u}_a \right) . \end{aligned}$$

Comparing the expressions of the 4-acceleration, $\dot{u}_a\equiv u^b\nabla _b u_a$ and the heat flux density $q^{(\phi )}_a$ leads to the identification

6 $$\begin{aligned} q_a^{(\phi )} = -\frac{ \sqrt{-\nabla ^c \phi \nabla _c \phi }}{ 8 \pi \phi } \, \dot{u}_a. \end{aligned}$$

In turn, comparison with the generalized Fourier law (5) leads to identifying a “temperature of gravity” $\mathcal {T}$, by

7 $$\begin{aligned} \mathcal {K} \mathcal {T} = \frac{ \sqrt{-\nabla ^c \phi \nabla _c \phi }}{ 8 \pi \phi } \end{aligned}$$

(see [3, 4, 10] for details). It is striking that $\mathcal{K}\mathcal{T}$ is positive-definite, which was not granted in this formal identification of quantities. A simple physical interpretation of $\mathcal {T}$ for theories described by the action (1) was elaborated in [4]. One finds that $\mathcal {T}=\dfrac{1}{\phi }=G_\textrm{eff}$, thus the effective temperature measures the strength of the gravitational interaction. Moreover, $\mathcal{K}\mathcal{T}$ vanishes in the GR limit where there is no $\phi$-fluid, namely if $\phi$ is constant and $G_\textrm{eff}$ is simply the gravitational constant *G*. We can thus identify GR with the $\mathcal{K}\mathcal{T}=0$ equilibrium state in this “thermodynamics of gravitational theories”, whereas scalar-tensor theories with an additional dynamical degree of freedom to those of GR represent non-equilibrium states with $\mathcal{K}\mathcal{T}>0$.

An effective heat equation for the $\phi$-fluid illustrating the behaviour of $\mathcal{K}\mathcal{T}$ with time can be found by differentiating (7). Taking $\dfrac{d}{d\tau }\equiv u^c\nabla _c$, we obtain

8 $$\begin{aligned} \frac{d(\mathcal{K}\mathcal{T})}{d\tau }=8\pi (\mathcal{K}\mathcal{T})^2-\Theta (\mathcal{K}\mathcal{T})+\frac{\Box \phi }{8\pi \phi } \,, \end{aligned}$$

where $\Theta = \nabla _c u^c$ is the expansion scalar of the effective fluid. This equation can be studied to understand whether the class of gravity theories (or a solution thereof) with a given $\mathcal{K}\mathcal{T}$ approaches or departs from the GR equilibrium and gives a precise meaning to the dissipative process leading from non-equilibrium to equilibrium in the thermodynamical analogy developed so far. The physical interpretation of this equation given in [4] for a simple case shows that, for example, $\mathcal{K}\mathcal{T}$ diverges at spacetime singularities and there are situations where the equilibrium state is never approached.

Equation (8) has two fixed points, $\mathcal{K}\mathcal{T}=0$ and $\mathcal{K}\mathcal{T}=\mathrm const.>0$. We explore both because, if stable, they could correspond to equilibrium states other than GR. Gravitational theories with non-dynamical scalar fields have been shown to recover $\mathcal{K}\mathcal{T}=0$ in [49], while a state with $\mathcal{K}\mathcal{T}=\mathrm const.$ that never approaches the GR equilibrium state was found in [33] to be metastable. Here, we complement this analysis by studying more stealth solutions and assessing their stability with a new, purely thermodynamical, criterion found as follows.

Equation (8) can be recast in a Klein–Gordon-like form as

9 $$\begin{aligned} \Box \phi -m_\textrm{eff}^2 \phi =0, \end{aligned}$$

where

10 $$\begin{aligned} m_\textrm{eff}^2 \equiv 8\pi \left[ \frac{d\left( \mathcal{K}\mathcal{T}\right) }{d\tau } - 8\pi \left( \mathcal{K}\mathcal{T}\right) ^2 + \Theta \, \mathcal{K}\mathcal{T} \right] \,. \end{aligned}$$

The effective mass $m_\textrm{eff}$ is clearly not a physical mass, but simply an effective quantity derived in the context of the thermodynamical analogy explained above. The sign of $m_\textrm{eff}^2$ can be used to construct a stability criterion, based on the need to avoid tachyonic instabilities. Indeed, we have *instability* if the square of the effective mass (that we call “thermal mass”) is $m_\textrm{eff}^2<0$ and *stability* if

11 $$\begin{aligned} m_\textrm{eff}^2 \ge 0. \end{aligned}$$

Since $\mathcal{K}\mathcal{T}$ is a scalar, this stability criterion is covariant and gauge-invariant. Of course, this notion of stability is only meaningful in the context of the thermodynamical analogy at hand to assess theories or solutions with a given $\mathcal{K}\mathcal{T}$, whose time evolution is described by (8). It is distinct from and unrelated to an assessment of the perturbative stability of the solution. This effective mass of scalar-tensor gravity differs from those explored in [52, 53].

It may seem odd that thermal stability reduces to avoiding tachyonic instabilities determined by an effective mass that depends on $\mathcal{K}\mathcal{T}$ and its derivative. However, this is exactly the same philosophy used to deduce the notorious Dolgov–Kawasaki stability criterion for metric *f*(*R*) gravity [50, 51]. Since the effective mass $m_\textrm{eff}$ is built only out of $\mathcal{K}\mathcal{T}$ and its time derivative and is deduced from the effective thermal description of scalar-tensor gravity, this stability criterion is definitely thermal (in the sense of said effective thermodynamics). It is made possible by the fact that an equation describing the approach to thermal equilibrium (or departure from it) exists in the theory. Near states of thermal equilibrium, it has essentially the same physical content as the effective heat equation.

The thermal stability criterion (11) is not particularly useful in the general thermodynamics of scalar-tensor gravity because one does not *a priori* know the quantities appearing in (10). However, if one wants to assess the stability *of specific solutions* (or classes of solutions) of the field equations, (11) is indeed suitable. This is the goal of the rest of this work. The criterion was used in [49] to study Nordström gravity, finding it unstable.

## Stealth solutions

The stealth solutions we are interested in here are special cases where Minkowski space results not from the absence of matter, but from a tuned balance between matter and the Brans–Dicke scalar or, in vacuo, between different terms in the scalar contribution to the stress-energy tensor. Stealth solutions like those studied in [34–37] are interesting since they show that Minkowski space is not necessarily devoid of matter, and the effect of gravitational coupling persists in the energy–momentum tensor even when this coupling is switched off.

Stealth solutions commonly encountered in the literature in the context of the scalar-tensor theory (1) are usually of two kinds:

1. $$g_{ab}=\eta _{ab} $$ and $$\phi =\phi _0 \, \text{ e}^{\alpha \, t} $$;
2. $$g_{ab}=\eta _{ab} $$ and $$\phi =\phi _0 \, | t |^{\beta } $$,

where $\eta _{ab}$ is the Minkowski metric in Cartesian coordinates, $\phi _0, \alpha , \beta$ are constants, and $\phi _0>0$ so that gravity is always attractive.

Differentiation yields

12 $$\begin{aligned} \dot{\phi } = \phi \times \left\{ \begin{aligned}&\alpha \, , \\&\frac{\beta }{t} \,,\; \; \text{ if } \; t\ne 0 \,, \end{aligned} \right. \end{aligned}$$

thus the requirement of future-directed scalar gradient translates into the conditions

13 $$\begin{aligned} \phi > 0 \quad \text{ and } \quad g_{ab} \nabla ^a \phi \, (\partial _t)^b < 0 \end{aligned}$$

or, for the specific scenarios above,

14 $$\begin{aligned} 0 > g_{ab} \nabla ^a \phi \, (\partial _t)^b= & {} g_{ab} \left( g^{a0} \dot{\phi } \right) \, {\delta ^b}_0 = g_{00} \, g^{00} \dot{\phi } = \dot{\phi } \nonumber \\= & {} \phi \times \left\{ \begin{aligned}&\alpha \,, \\&\frac{\beta }{t} \,\, \; \text{ if } \; t \ne 0 \,. \end{aligned} \right. \end{aligned}$$

Thus, enforcing the future orientation of the scalar field gradient, we shall restrict to cases that satisfy the conditions

1. $$\alpha < 0$$;
2. $$\beta <0 $$  if $$t>0$$ or  $$\beta >0$$  if $$ t<0$$.

In the first case

15 $$\begin{aligned} \mathcal{K}\mathcal{T} = \frac{ \sqrt{-\nabla ^c \phi \nabla _c\phi }}{8\pi \phi } = \frac{|\alpha |}{8\pi } =\text{ const. }>0 \,, \end{aligned}$$

which means that this solution never approaches the GR equilibrium state. If we now consider its stability from the point of view of first-order thermodynamics, we see that the effective mass is constant and given by

16 $$\begin{aligned} m_\textrm{eff}^2= & {} \frac{\Box \phi }{\phi } = \frac{\partial ^{\mu }\partial _{\mu }\phi }{\phi } \nonumber \\= & {} \frac{\partial ^{\mu } \left( \alpha \, {\delta ^0}_{\mu } \phi \right) }{\phi } = -\alpha ^2 <0 , \end{aligned}$$

which makes this stealth solution *unstable*. A stealth solution of this type was assessed in [33] with the gauge-invariant criterion for cosmological perturbations and shown to be a metastable state.

In the second case, $\beta =1$ and $\beta =2$ are the most relevant situations encountered in the literature. Therefore, according to our conventions, in order to have $G_\textrm{eff} = \phi ^{-1}>0$ and $u^a = \nabla ^a \phi / \sqrt{-\nabla ^c\phi \nabla _c\phi }$ future-oriented, it must be $\phi _0>0$ in conjunction with $t<0$ if $\beta > 0$.

Then, if $\beta > 0$ the effective gravitational coupling behaves as

17 $$\begin{aligned} G_\textrm{eff}=\frac{1}{\phi } = \frac{1}{\phi _0 \, |t|^\beta } \rightarrow +\infty \quad \text{ as } \; t\rightarrow 0^{-} \, , \end{aligned}$$

the effective temperature of gravity (7) is

18 $$\begin{aligned} \mathcal{K}\mathcal{T} = \frac{ \beta }{8\pi |t|} \rightarrow +\infty \quad \text{ as } \; t\rightarrow 0^{-} \, , \end{aligned}$$

and the effective mass reads

19 $$\begin{aligned} m_\textrm{eff}^2 = \frac{\Box \phi }{\phi } = - \frac{\beta (\beta - 1)}{t^2} \, . \end{aligned}$$

If $\beta =1$, we get $m_\textrm{eff}^2 = 0$. Therefore this constant “mass” solution is *marginally stable*. As $t\rightarrow 0^{-}$, we approach a singularity of the theory where $G_\textrm{eff}\rightarrow + \infty$, $\mathcal{K}\mathcal{T} \rightarrow +\infty$, gravity becomes infinitely strong and deviates from GR drastically. Indeed, nothing could be further from a GR situation than infinitely strong gravity with Minkowski spacetime! This solution matches the idea that singularities are “hot” in the sense of the thermodynamics of scalar-tensor gravity [3, 4]. This situation is stable according to the thermal stability criterion (11). Hence, barring instabilities of a different nature, one expects this behaviour to occur in nature if singularities are present. The implication is that the GR equilibrium state is not always approached and gravity indeed departs from GR near singularities. Of course, the final theory of gravity should remove singularities, but it is clear that scalar-tensor gravity is not this final theory since it does contain spacetime singularities and singularities of $G_\textrm{eff}$.

The situation where $\beta =2$, exemplified in Sect. 3.2, entails $m_\textrm{eff}^2 = - 2/t^2 <0$, meaning *instability* from the thermal point of view, while $\mathcal{K}\mathcal{T} = 1/4\pi |t|$ and $G_\textrm{eff} = 1/\phi _0 t^2$ both diverge as $t\rightarrow 0^{-}$, thus departing from GR at this singularity of $G_\textrm{eff}$. In our formalism the $t>0$ branch of the solution is not meaningful.

Most exact solutions of Brans-Dicke theories in cosmology exhibit the power-law behaviour $\phi =\phi _0\,t^{\beta }$ [54], such as those found by O’Hanlon and Tupper [55] and Nariai [56, 57]. These were studied from the point of view of first-order thermodynamics in [14], and in Sects. 3.1 and 3.2 we consider two degenerate cases of such solutions that reduce to a Minkowski background with a non-trivial scalar field profile.

Other types of stealth solutions with Minkowski metric and non-trivial scalar include those found for a nonminimally coupled $\phi$ [35], where the field is inhomogeneous, wave-like, and does not gravitate. Their stability was studied in [58] using the Bardeen–Ellis–Bruni–Hwang gauge-invariant formalism for cosmological perturbations [22–26], showing mixed stability results depending on the specific choice of parameters. These solutions either do not correspond to future-oriented four-velocity $u^c$, or are very cumbersome to discuss because $\nabla ^a\phi$ is timelike only in very restricted spacetime regions and for special combinations of their parameters. Therefore, they will not be examined here.

### O’Hanlon & Tupper (OHT) solution with $\omega =0$

The O’Hanlon & Tupper spatially flat FLRW solution of Brans–Dicke cosmology is obtained from the action (1) for $\omega =\text{ const. }>-3/2$ and $\omega \ne -4/3$ and $V=0$ [55]. The scale factor and scalar field read

20 $$\begin{aligned} a(t)= & {} a_0 \left( \frac{t}{t_0} \right) ^{q_{\pm }} , \end{aligned}$$

21 $$\begin{aligned} \phi (t)= & {} \phi _0 \left( \frac{t}{t_0} \right) ^{s_{\pm }} , \end{aligned}$$

with

22 $$\begin{aligned} q_{\pm }= & {} \frac{\omega }{3(\omega +1) \mp \sqrt{ 3(2\omega +3)}} , \end{aligned}$$

23 $$\begin{aligned} s_{\pm }= & {} \frac{ 1 \mp \sqrt{3(2\omega +3)}}{3\omega +4} , \end{aligned}$$

and $3q_{\pm }+s_{\pm }=1$. This solution has a “hot” singularity at $t\rightarrow 0^+$, where Brans-Dicke theory departs from the GR behaviour. Although the value $\omega =0$ was not contemplated in [55], it is straightforward to check that it corresponds to a Minkowski space solution of the equations of vacuum Brans-Dicke cosmology with $V=0$, $q=0$, $a(t)=1$, and linear scalar field $\phi (t) =\phi _0 \, t$ (choosing $t_0=1$ for convenience). This is a *bona fide* stealth solution, which could have been introduced in Ref. [55] long before solutions with this name were noticed and appreciated [15, 34–46]. In order for the four-velocity to be future-oriented and for $G_\textrm{eff}$ to be positive, it must be $\phi _0<0$ and $t<0$. This situation is akin to case 2. with $\beta = 1$ considered above, hence the $\omega =0$ O’Hanlon & Tupper solution turns out to be *marginally stable* according to the thermal stability criterion,^1^ This universe has

24 $$\begin{aligned} \mathcal{K}\mathcal{T}=\frac{1}{8\pi |t|} \rightarrow +\infty \end{aligned}$$

as $t\rightarrow 0^{-}$, deviating from GR.

### Nariai solution with $\omega =-1/2$

The Nariai solution [56, 57] is a particular power-law solution for a $K=0$ FLRW universe with perfect fluid matter that has $P=\left( \gamma -1 \right) \rho$ (with $\gamma =$ const.), $V(\phi )=0$ and $\omega \ne -4 \left[ 3\gamma \left( 2-\gamma \right) \right] ^{-1}<0$. Here we are interested in a cosmological constant fluid with $\gamma =0$, $P^\mathrm {(m)}=-\rho ^\mathrm {(m)}$, and

25 $$\begin{aligned} a(t)= & {} a_0 \left( 1+\delta t \right) ^{\omega +1/2} , \end{aligned}$$

26 $$\begin{aligned} \phi (t)= & {} \phi _0 \left( 1+\delta t \right) ^2 , \end{aligned}$$

27 $$\begin{aligned} \delta= & {} \left[ \frac{32\pi \rho _0}{\phi _0} \frac{1}{\left( 6\omega +5 \right) \left( 2\omega +3 \right) }\right] ^{1/2} . \end{aligned}$$

This solution is an attractor in phase space and was used in the extended inflationary scenario [59, 60]. For $\omega =-1/2$, $\delta =\sqrt{ 8\pi \rho _0/\phi _0}$, the scale factor is constant and $H=0$, making this a Minkowski stealth solution with non-trivial (polynomial) scalar field profile. It is a straightforward generalisation of the type 2. stealth solutions described above.^2^ It must be $\phi _0>0, \left( 1+\delta t \right) <0$ and

28 $$\begin{aligned} \mathcal {K}\mathcal {T} = \frac{\delta }{4\pi |1+\delta t|} \rightarrow +\infty \end{aligned}$$

as $\left( 1+\delta t\right) \rightarrow 0^{-}$. In the far past $t \rightarrow - \infty$, $\mathcal {K}\mathcal {T}\rightarrow 0$ and GR is approached, but the instability prevents this state from being an equilibrium alternative to GR. In fact, the thermal stability criterion yields

29 $$\begin{aligned} m^2_\textrm{eff}=\frac{\Box \phi }{\phi }= - \, \frac{2 \delta ^2}{\left( 1+\delta t \right) ^2}<0 \end{aligned}$$

and this solution is thermally *unstable*.

## de Sitter space solutions

Other common solutions of scalar-tensor gravity are de Sitter ones with line element

30 $$\begin{aligned} ds^2 =-dt^2 +a_0^2 \, \text{ e}^{2H_0 t} \left( dx^2 +dy^2 +dz^2 \right) \end{aligned}$$

in comoving coordinates, with scale factor $a(t)=a_0 \, \text{ e}^{H_0t}$, where $a_0, H_0$ are constants.

In GR with a minimally coupled scalar field as the only matter source, the only possible de Sitter spaces are obtained for a constant scalar field, $\left( H, \phi \right) =\left( H_0, \phi _0 \right)$, with both $H_0$ and $\phi _0$ constant. In spatially flat FLRW cosmology, the independent dynamical variables are^3^$\left( H, \phi \right)$ and the phase space is a 2-dimensional subset of the 3-dimensional space $\left( H, \phi , \dot{\phi } \right)$ identified by the Hamiltonian constraint. This 2-dimensional subset is analogous to an energy surface in point particle mechanics [61, 62]. The points $\left( H_0, \phi _0 \right)$ are then all the equilibrium points of the dynamical system.

For spatially flat FLRW universes in scalar-tensor cosmology, the independent variables are still *H* and $\phi$ and there can be fixed points $\left( H_0, \phi _0 \right)$ of this dynamical system. The structure of the phase space and the fixed points for specific scalar-tensor theories are discussed extensively in [47, 61, 63], respectively. Gauge-invariant criteria for the stability of these de Sitter fixed points (and of their degenerate Minkowski cases) are given in [64–68]. In addition to de Sitter fixed points, in scalar-tensor cosmology there can be de Sitter spaces with non-constant scalar field, usually exponential or power-law in time. Since these are only admissible in modified gravity and not in GR, they are interesting for first-order thermodynamics. Degenerate cases of such de Sitter solutions can reproduce Minkowski space with a non-trivial scalar field and are therefore another kind of stealth solutions similar to those of the previous section.

### de Sitter solutions of scalar-tensor gravity

This type of solution, known in many scalar-tensor theories, is found starting from the action (1) and reads

31 $$\begin{aligned} H= & {} H_0 = \text{ const. } , \end{aligned}$$

32 $$\begin{aligned} \phi (t)= & {} \phi _0 \, \text{ e}^{\alpha \, t} , \end{aligned}$$

with $\phi _0$ a positive constant. The constants $H_0$ and $\alpha$ are related to the parameters of the specific scalar-tensor theory. Although these solutions have been known for a long time, here we consider them from the novel point of view of scalar-tensor thermodynamics.

In order to get a future-directed four-velocity of the effective $\phi$-fluid and an attractive gravitational interaction we need to require, again, that

33 $$\begin{aligned} \phi > 0 \quad \text{ and } \quad g_{ab} \nabla ^a \phi \, (\partial _t)^b < 0 \, , \end{aligned}$$

which implies $\phi _0 > 0$ and $\alpha < 0$.

We have (as in (15))

34 $$\begin{aligned} \mathcal{K}\mathcal{T}= \frac{|\alpha |}{8\pi }= \text{ const. } \end{aligned}$$

and this solution remains away from the zero-temperature GR state of equilibrium at all times. Is it thermally stable? We find

35 $$\begin{aligned} m_\textrm{eff}^2= & {} \frac{\Box \phi }{\phi } = \frac{ -\left( \ddot{\phi }+3H_0 \dot{\phi } \right) }{\phi } = -\alpha \left( \alpha +3H_0 \right) \nonumber \\= & {} |\alpha |\left( 3H_0-|\alpha | \right) ; \end{aligned}$$

therefore, we have *stability* for $3H_0 \ge |\alpha |$ and *instability* for $|\alpha | > 3H_0$.

In particular, it is clear that exponentially contracting FLRW universes ($H_0<0$) are always unstable. This conclusion, obtained with simple considerations in scalar-tensor thermodynamics, matches the result found in the literature on scalar-tensor cosmology [64] with a dynamical systems analysis which requires the complete specification of the theory.

#### Kolitch solutions of vacuum Brans–Dicke cosmology with cosmological constant

Kolitch [69] found solutions of vacuum Brans–Dicke cosmology with positive cosmological constant $\Lambda$, equivalent to the linear potential $V(\phi ) = 2\Lambda \phi$. These solutions were previously noted in [70, 71] and read

36 $$\begin{aligned} a(t)= & {} a_0 \exp \left[ \pm \left( \omega +1 \right) \sqrt{ \frac{2\Lambda }{(2\omega +3)(3\omega +4)}} \, t \right] , \end{aligned}$$

37 $$\begin{aligned} \phi (t)= & {} \phi _0 \exp \left[ \pm \sqrt{ \frac{2\Lambda }{(2\omega +3)(3\omega +4)}} \, t \right] . \end{aligned}$$

For $\omega =-1$, they reduce to the stealth solution with

38 $$\begin{aligned} H=0 \,, \quad \quad a(t)=1 \,, \quad \quad \phi (t)=\phi _0 \, \text{ e}^{ \pm \sqrt{2\Lambda } \, t, } \end{aligned}$$

where, again, we must choose the lower sign to have a future-oriented four-velocity. This solution deviates from GR at all times since $\mathcal{K}\mathcal{T}=\text{ const. }>0$, but it corresponds to $m_\textrm{eff}^2 =-\alpha ^2 <0$ and is *unstable*. Its stability has also been studied with respect to both homogeneous and inhomogenous metric perturbations in [58], where the solution with the upper sign is found to be stable and the one with the lower sign unstable. However, the solution with the upper sign cannot be analysed in the framework of scalar-tensor thermodynamics since it entails a past-oriented $\nabla ^a \phi$.

Let us consider now the de Sitter spaces (36), (37) for $\omega \ne -1$: taking the lower sign we have

39 $$\begin{aligned} H_0 = -\left( \omega +1\right) \sqrt{ \frac{2\Lambda }{(2\omega +3)(3\omega +4)}} \equiv - \left( \omega +1\right) \textrm{C} \end{aligned}$$

and

40 $$\begin{aligned} \alpha =- \sqrt{\frac{2\Lambda }{(2\omega +3)(3\omega +4)}} \equiv - \textrm{C} \, , \end{aligned}$$

where $\textrm{C}$ is a positive real constant if $\omega < -3/2$ and $\omega > -4/3$. Therefore, the effective mass reads

41 $$\begin{aligned} m_\textrm{eff}^2= & {} |\alpha |\left( 3H_0-|\alpha | \right) = -\textrm{C}^2 \left( 3 \omega + 4 \right) \end{aligned}$$

Then, if $\omega < -3/2$ we have an expanding de Sitter universe which is thermodynamically *stable*, although the scalar field for such values of the coupling is phantom and therefore suffers from different types of instabilities [72]. Other configurations are otherwise *unstable*.

#### O’Hanlon & Tupper solution in the $\omega \rightarrow -4/3$ limit

It is often mentioned in the literature that the O’Hanlon & Tupper solution (20)–(23) approaches de Sitter space in the limit $\omega \rightarrow -4/3$, recovering

42 $$\begin{aligned} a(t)= & {} a_0 \exp {(H_0\,t)} , \end{aligned}$$

43 $$\begin{aligned} \phi (t)= & {} \phi _0\exp {(-3H_0\,t)} , \end{aligned}$$

with $H_0$ a positive constant. Technically, this statement is not accurate since the above result is recovered by simultaneously choosing the values $q_+$ and $s_-$ of the exponents, which correspond to two distinct solutions. However, the solution above is the only de Sitter one for flat FLRW and vacuum [72]. Given that $\alpha <0$, the velocity of the scalar field fluid is future-oriented and $3H_0-|\alpha |=0$, so this solution is *marginally stable* according to the thermal criterion.

This solution describes expanding universes for which the effective fluid four-velocity is only future-oriented. These expanding universes are unstable with respect to tensor modes, as can be concluded using the Bardeen–Ellis–Bruni gauge-invariant formalism for cosmological perturbations [22–26] in Hwang’s version adapted to modified gravity [27–32]. The relevant equations are summarized in Appendix A. We only need Eq. (A.14) for the gauge-invariant variable $H_T$ associated with the tensor modes which, in the background (42) and (43), becomes

44 $$\begin{aligned} \ddot{H}_T +\left( 3H+ \frac{\dot{\phi }}{\phi } \right) \dot{H}_T + \frac{k^2}{a^2(t)} \, H_T =0 \,, \end{aligned}$$

where *k* is the mode’s momentum and the coefficients are given by the unperturbed *a*(*t*) and $\phi (t)$, which yields $3\,H+\dot{\phi }/\phi =0$ to zero order. With $H_0>0$, the asymptotic equation at late times $t\rightarrow +\infty$ reduces to

45 $$\begin{aligned} \ddot{H}_T +\frac{k^2}{a^2} \, H_T \simeq \ddot{H}_T=0 \,, \end{aligned}$$

with linear solution $H(t)= \alpha \, t+ \text{ const. }$ The tensor perturbation diverges and this universe is *unstable*.

### Constant curvature spaces in *f*(*R*) gravity

Metric *f*(*R*) gravity is a subclass of scalar-tensor theories described by the action

46 $$\begin{aligned} S_{f(R)}=\frac{1}{16\pi } \int d^4 x \sqrt{-g} f(R) +S^\mathrm {(m)} \end{aligned}$$

and is equivalent [73–75] to a Brans–Dicke theory with $\phi = f'(R)$ (a prime denotes differentiation with respect to *R*), $\omega =0$, and the potential

47 $$\begin{aligned} V(\phi ) =Rf'(R)-f(R) \Bigg |_{f'(R)=\phi } \,. \end{aligned}$$

Assuming that $\nabla ^c R$ is timelike and future-oriented, the effective dissipative fluid associated with *f*(*R*) gravity has [3]

48 $$\begin{aligned} \mathcal{K}\mathcal{T} = \frac{ f''(R) \sqrt{ -\nabla ^c R \nabla _c R}}{8\pi f'(R) } \,, \end{aligned}$$

where it is required that $f'(R)>0$ in order for the effective gravitational coupling $G_\textrm{eff} =1/\phi$ to be positive and for the graviton to carry positive kinetic energy, while $f''(R) \ge 0$ is required for local stability [51] (here $\nabla ^c \phi$ is timelike and future-oriented if $\nabla ^c R$ is).

The fact that the effective Brans–Dicke scalar field $\phi$ in *f*(*R*) gravity is tied so intimately with the Ricci scalar makes all constant curvature spaces in these theories zero-temperature states indistinguishable from GR, because this means that $\phi =f'(R)=$ const. and $\nabla _c \phi$ vanishes identically, together with $\mathcal{K}\mathcal{T}$. Furthermore, these states are *(marginally) stable* in our thermal sense because $\Box \phi =0$ and the effective mass $m_\textrm{eff}^2= \Box \phi /\phi$ also vanishes identically.

The condition $m^2_\textrm{eff} \ge 0$ for the thermal stability of *f*(*R*) gravity does not coincide with the stability condition of de Sitter space with respect to first order local perturbations, obtained in a gauge-invariant way ([68] and references therein),

49 $$\begin{aligned} \left( f_0' \right) ^2-2f_0 f_0'' \ge 0 \,, \end{aligned}$$

where a zero subscript denotes a quantity evaluated on the de Sitter background. Therefore, the thermal stability condition $m^2_\textrm{eff} \ge 0$ does not necessarily coincide with other stability notions, as could be expected. Indeed, also in Newtonian systems and in GR one has different notions of stability (thermal, dynamical, *etc.*) and the thermodynamics of modified gravity evidently cannot account for all possible notions of stability.

**Table 1:** Summary of the analytical solutions studied and their thermal stability

Solution	Type	Thermal stability
OHT $\omega =0$	Minkowski stealth	Marginally stable (departs from GR as $t\rightarrow 0^{-}$)
Nariai $\omega =-1/2$	Minkowski stealth	Unstable
Kolitch $\omega =-1$	Minkowski stealth	Unstable
Kolitch $\omega <-3/2$	de Sitter	Stable (but $\phi$ phantom)
OHT $\omega \rightarrow -4/3$	de Sitter	Marginally stable
*f*(*R*) gravity	(Anti-)de Sitter, Minkowski	Marginally stable

## Conclusions

In the context of the new thermodynamics of scalar-tensor gravity, the role of GR as the thermal state of equilibrium and its stability are physically significant. The thermal description of scalar-tensor gravity connects to the broad idea of gravity being emergent rather than fundamental, setting it apart from the three other interactions we know. The idea of alternative gravity as a thermal non-equilibrium state, first proposed in Jacobson’s thermodynamics of spacetime, adds to this picture. In spite of the numerous articles citing Jacobson’s seminal papers, this subsequent body of work on spacetime thermodynamics has never been able to identify the “temperature of gravity” (or another order parameter) and to produce equations describing the approach to equilibrium (or lack thereof). Our new (and completely different) thermodynamics of scalar-tensor gravity does that. Then, it becomes important to identify states of thermal equilibrium and their stability, which is done here.

Specifically, the significance of the new thermal stability criterion derived here lies in the fact that it can reject certain solutions of scalar-tensor gravity that, although mathematically possible, cannot occur in nature because they are unstable. Our stability criterion expresses thermal stability because it is derived from the equation describing the approach to thermal equilibrium or the departures from it in scalar-tensor gravity. Near thermal equilibrium states, this criterion expresses the physical content of the equation describing the approach to equilibrium in a way that makes it easier and practical to assess the stability of analytical solutions of the scalar-tensor field equations.

In this work, we studied the states of gravity corresponding to $\mathcal{K}\mathcal{T}=\mathrm{const.}$, which are fixed points of the effective heat equation describing the approach to (or departure from) equilibrium (8), in the context of first-order thermodynamics [4]. These states, away from the GR equilibrium, correspond to different types of stealth solutions, which are not admitted by the Einstein equations and are thus a signature of alternative gravity [15, 34–46].

Specifically, we studied the scalar field profiles 1. $\phi =\phi _0 \, \text{ e}^{\alpha \, t}$ (with $\alpha <0$) and 2. $\phi =\phi _0 \, | t |^{\beta }$ (with $t>0, \beta <0$ or with $t<0, \beta >0$), common in the literature. The first case has $\mathcal{K}\mathcal{T}= \text{ const. }>0$, which would correspond to a state of equilibrium at positive temperature. However, this state is unstable according to the thermal criterion (11). This criterion does not necessarily go hand-in-hand with other stability criteria, which should not come as a surprise, since a physical system can be subject to instabilities of different nature, with different time scales. Sometimes instability in the thermal sense (11) is accompanied by instability with respect to gravitational perturbations; however, this coincidence should not always be expected.

In any case, stable equilibrium states of gravity with $\mathcal{K}\mathcal{T}=\mathrm const.$ either do not exist or are fragile and easily destroyed by perturbations (i.e., metastable).

Stealth solutions with a linear scalar field profile, as in the second case, require caution because, combining the requirements that $G_\textrm{eff}>0$ and that the effective $\phi$-fluid four-velocity $u^a$ be future-oriented (essential when discussing dissipation associated with an arrow of time), one finds a singularity of the effective gravitational coupling at $t=0$, which can justly be regarded as a “thermodynamical” singularity of scalar-tensor gravity. These spaces are stable according to the thermal criterion and are not destroyed by perturbations (as far as scalar-tensor gravity applies), but $\mathcal{K}\mathcal{T}$ diverges at this singularity, as it does in ordinary spacetime singularities, signalling a drastic deviation from GR predicted in [3, 4]. This result reinforces the idea that gravity strongly deviates from GR at singularities, but now the concept of “thermodynamical singularity” is extended to include also singularities of the effective gravitational coupling $G_\textrm{eff}$. These considerations, of course, do not solve the spacetime singularity problem of relativistic gravity; the temperature $\mathcal {T}$ introduced by scalar-tensor thermodynamics is relative to the GR state and measures the distance of the actual state of gravity from the GR state of equilibrium at $\mathcal{K}\mathcal{T}=0$, which is still affected by the spacetime singularity problem.

The realization that stealth solutions of scalar-tensor gravity are often degenerate cases of de Sitter universes with non-constant Brans–Dicke-like scalar field prompts the consideration of these spaces (Sect. 4). It is intriguing that the cosmic no-hair theorem (when valid) can be seen in a new light from the point of view of scalar-tensor thermodynamics. (The validity, or lack thereof, of cosmic no-hair in various scalar-tensor gravities will be examined from the thermal point of view in future work). On the one hand, de Sitter spaces with constant scalar field can be attractors of the cosmological dynamics (even starting with anisotropic Bianchi models) but, when $\phi$ is constant, $\mathcal{K}\mathcal{T}$ vanishes and gravity reduces to its zero-temperature GR state of equilibrium.^4^ On the other hand, de Sitter spaces with non-constant scalar field are known to occur in various scalar-tensor gravities (where they are not attractors of the cosmological dynamics) but are impossible in GR and are a signature of alternative gravity. In this sense, they can be regarded as generalizations of stealth solutions [78] and as such they were studied here from the point of view of first-order thermodynamics.

The results obtained for the solutions of scalar-tensor gravity analyzed here are summarized in Table 1. Overall, the two general principles of first-order thermodynamics of scalar-tensor gravity are confirmed: (i) gravity deviates wildly from GR near spacetime singularities and near singularities of the gravitational coupling; (ii) the convergence of gravity to GR at late times is marked by $\mathcal {K}\mathcal {T} \rightarrow 0$. No states of equilibrium $\mathcal {K}\mathcal {T}=$ const. other than GR (corresponding to $\mathcal {K}\mathcal {T}=0$) have been found here, except for solutions that are unstable according to various criteria and are, therefore, physically irrelevant. This result reinforces the special role of general relativity as an equilibrium state in the landscape of gravity theories, seen through the lens of first-order thermodynamics. The results above will be useful in the following developments of the first-order thermodynamical formalism.

## Data Availability

This manuscript has no associated data or the data will not be deposited. [Authors’ comment: There are no data associated with this article because of its theoretical and mathematical nature.]

## Appendix A: Gauge-invariant perturbations for scalar-tensor cosmology

Consider the modified gravity described by the action

A.1 $$\begin{aligned} S=\int d^{4}x\,\sqrt{-g}\,\left[ \frac{f(\phi ,R)}{2} -\frac{ \bar{\omega }(\phi )}{2} \, \nabla ^c \phi \nabla _c \phi -V\left( \phi \right) \right] \end{aligned}$$

and a spatially flat unperturbed FLRW universe with line element

A.2 $$\begin{aligned} ds^2=-dt^2+a^2(t) \left( dx^2+dy^2 +dz^2 \right) \,. \end{aligned}$$

The unperturbed field equations are

A.3 $$\begin{aligned} H^{2}= & {} \frac{1}{3F} \left( \frac{ \bar{\omega }}{2} \, \dot{\phi }^{2} +\frac{RF}{2}-\frac{f}{2}+V-3H\dot{F}\right) \,, \end{aligned}$$

A.4 $$\begin{aligned} \dot{H}= & {} -\frac{1}{2F}\left( \bar{\omega } \, \dot{\phi }^{2} +\ddot{F}-H\dot{F}\right) \,, \end{aligned}$$

A.5 $$\begin{aligned} \ddot{\phi }+ & {} 3H\dot{\phi }+\frac{1}{2 \bar{\omega }} \left( \frac{d \bar{\omega }}{d\phi } \, \dot{\phi }^{2}-\frac{\partial f}{ \partial \phi }+2 \, \frac{dV}{d\phi }\right) =0\,, \end{aligned}$$

where an overdot denotes differentiation with respect to the comoving time *t*, $H\equiv \dot{a}/a$ is the Hubble function, and $F\equiv \partial f/\partial R$. Quantities denoted with $A,\, B,\, H_{L}$, and $H_{T}$ define the metric perturbations in the Bardeen–Ellis–Bruni–Hwang formalism [22–26] according to

A.6 $$\begin{aligned} g_{00}= & {} -a^{2}\left( 1+2AY\right) \,, \end{aligned}$$

A.7 $$\begin{aligned} g_{0i}= & {} -a^{2}BY_{i}\,, \end{aligned}$$

A.8 $$\begin{aligned} g_{ij}= & {} a^{2}\left[ h_{ij}\left( 1+2H_{L}\right) +2H_{T}Y_{ij}\right] , \end{aligned}$$

where $h_{ij}$ is the 3-metric of the unperturbed FLRW space seen by the comoving observer, the scalar harmonics *Y* satisfy the eigenvalue problem $\bar{\nabla }_{i}\bar{\nabla }^i Y=-k^{2}Y$ with eigenvalue *k*, and $\bar{\nabla _{i}}$ is the covariant derivative operator of $h_{ij}$. The vector and tensor harmonics $Y_{i}$ and $Y_{ij}$ satisfy

A.9 $$\begin{aligned} Y_{i}= & {} -\frac{1}{k} \, \bar{\nabla }_{i}Y\,, \end{aligned}$$

A.10 $$\begin{aligned} Y_{ij}= & {} \frac{1}{k^2} \, \bar{\nabla }_{i} \bar{\nabla }_{j}Y+\frac{1}{3}Yh_{ij}\,. \end{aligned}$$

A.11 $$\begin{aligned} \Phi _{H}= & {} H_{L}+\frac{H_{T}}{3} +\frac{\dot{a}}{k} \left( B -\frac{a}{k}\,\dot{H}_{T}\right) , \end{aligned}$$

A.12 $$\begin{aligned} \Phi _{A}= & {} A\!+\!\frac{\dot{a}}{k}\left( B \!-\!\frac{a}{k}\,\dot{H}_{T}\right) \!+\!\frac{a}{k}\left[ \dot{B}-\frac{1}{k} \left( a\dot{H}_{T}\right) \dot{} \,\right] , \end{aligned}$$

are the Bardeen gauge-invariant potentials [22],

A.13 $$\begin{aligned} \Delta \phi =\delta \phi +\frac{a}{k} \, \dot{\phi }\left( B -\frac{a}{k} \, \dot{H}_{T}\right) \end{aligned}$$

is the Ellis–Bruni variable [23, 24], and similar relations define the other gauge-invariant variables $\Delta f,\,\Delta F,$ and $\Delta R$. We refer the reader to Refs. [27–32] for the complete set of equations for the gauge-invariant perturbations. Here we only need the equation for the tensor modes

A.14 $$\begin{aligned} \ddot{H}_T +\left( 3H+ \frac{\dot{F}}{F} \right) \dot{H}_T +\frac{k^2}{a^2} \, H_T=0 \,, \end{aligned}$$

which is used in Sect. 4.

## Footnotes

1 In the analysis at the beginning of Sect. 3, we conventionally denoted $\phi (t) =\phi _0 \, |t|^\beta$ with $\phi _0>0$. In this section we instead employ the usual notation that can be found in the literature, i.e. $\phi (t) =\phi _0 \, t^\beta$, where $\phi _0$ and *t* can both be either positive or negative, provided that $\phi$ remains positive.

2 Here again we implicitly adapted our notation to the one which is typically employed in the literature. See footnote 1.

3 In the field equations for spatially flat FLRW universes, the scale factor only appears in the combination $H \equiv \dot{a}/a$.

4 Indeed, de Sitter spaces with constant scalar field are common attractors in GR according to Wald’s theorem ([76], see [77] for a review).
